# 2286. A comparison of SARS-CoV-2 wastewater-based surveillance in a sentinel community versus a large metropolitan sewershed in Massachusetts, 2020-2023

**DOI:** 10.1093/ofid/ofad500.1908

**Published:** 2023-11-27

**Authors:** Tyler Brown, Sowmya R Rao, Daniel Begemann, Flor Amaya, Barry Keppard, Amir Mohareb, Samantha Bates, Cristina Alonso, Katherine M Jia, Julie H Levison, Edward T Ryan, Regina C LaRocque

**Affiliations:** Boston University School of Medicine, Boston, Massachusetts; Boston University, Boston, Massachusetts; Center of Complex Interventions, Boston, Massachusetts; City of Chelsea, Chelsea, Massachusetts; Metropolitan Area Planning Council, Boston, Massachusetts; Massachusetts General Hospital Infectious Diseases Division, Boston, Massachusetts; Center of Complex Interventions, Boston, Massachusetts; Center of Complex Interventions, Boston, Massachusetts; Harvard T.H. Chan School of Public Health, Boston, Massachusetts; Massachusetts General Hospital and Harvard Medical School, Boston, Massachusetts; Massachusetts General Hospital, Boston, MA; Massachusetts General Hospital, Boston, MA

## Abstract

**Background:**

Many wastewater-based surveillance (WBS) programs for COVID-19 sample from distal endpoints of sewage treatment systems (for example, sewage treatment plants). SARS-CoV-2 RNA measurements from these endpoints represent highly aggregated cross-sections from entire sewage systems, and are inherently unable to capture local variation in COVID-19 disease activity. Importantly, using highly aggregated samples from sewage system endpoints may limit the utility of SARS-CoV-2 RNA measurements as a leading indicator for forecasting applications. Geographically-focused, hyperlocal sampling in communities with more intense and/or earlier COVID-19 epidemic activity is a promising strategy for enhancing the public health value of WBS.

Chelsea versus Boston-wide SARS-CoV-2 wastewater RNA concentration over time

**Figure:**
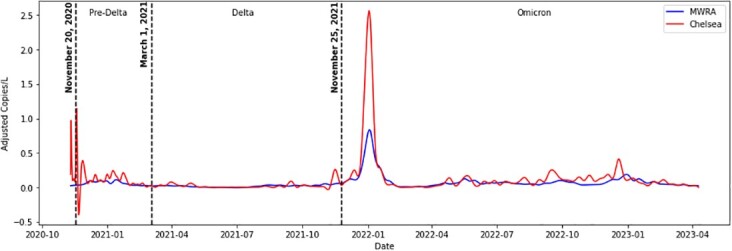

**Methods:**

We examined SARS-CoV-2 RNA data in Chelsea, Massachusetts, a Boston-area community that has experienced disproportionately high COVID-19 disease burden across all phases of the epidemic thus far. Between November 2020 and April 2023, we collected biweekly samples from four local sewershed locations in Chelsea, which were analyzed for SARS-CoV-2 RNA concentration by BioBot Analytics. We compared these local measurements to those for identically analyzed samples collected at a distal sewer system endpoint (Massachusetts Water Resource Authority Deer Island Treatment Plant), for the same time points over the same collection period.

**Results:**

Local sewershed SARS-CoV-2 RNA concentrations in Chelsea were consistently higher across all three major epidemic phases (pre-Delta, Delta, and Omicron) analyzed in our dataset. During the onset of the Delta wave, SARS-CoV-2 RNA concentrations in Chelsea increased earlier than Boston-wide levels measured at the sewer system endpoint.

**Conclusion:**

Local wastewater sampling can facilitate pre-emptive and tailored public health responses to more effectively counter the burden of SARS-CoV-2 in disproportionately affected communities. Sampling in these “sentinel” communities may improve the utility of SARS-CoV-2 wastewater RNA concentrations as a leading indicator for COVID-19 forecasting applications.

**Disclosures:**

**Julie H. Levison, MD, MPhil, MPH**, eMED, LLC.: Advisor/Consultant

